# Safety Profile of Lutein- Versus Triamcinolone Acetonide–Based Vitreous Staining

**DOI:** 10.1167/tvst.12.1.5

**Published:** 2023-01-04

**Authors:** Francesca Lazzara, Federica Conti, Mariantonia Ferrara, Myrta Lippera, Michele Coppola, Settimio Rossi, Filippo Drago, Claudio Bucolo, Mario R. Romano

**Affiliations:** 1 Department of Biomedical and Biotechnological Sciences, School of Medicine, University of Catania, Catania, Italy; 2 Manchester Royal Eye Hospital, Manchester University Hospitals NHS Foundation Trust, Manchester, UK; 3 Department of Biomedical Sciences, Humanitas University, Pieve Emanuele, Italy; 4 Department of Ophthalmology, San Gerardo Hospital, Monza, Italy; 5 Multidisciplinary Department of Medical, Surgical and Dental Sciences, University of Campania Luigi Vanvitelli, Naples, Italy; 6 Center for Research in Ocular Pharmacology–CERFO, University of Catania, Catania, Italy; 7 Eye Center, Humanitas Gavazzeni-Castelli, Bergamo, Italy

**Keywords:** lutein, triamcinolone, epiretinal membrane, internal limiting membrane, vitreoretinal pathologies

## Abstract

**Purpose:**

To assess the safety profile of a new lutein-based vitreous dye (LB-VD) formulation compared with various triamcinolone acetonide (TA) formulations with and without subsequent exposure to perfluorodecalin (PFD) in vitro.

**Methods:**

Human adult retinal pigment epithelial cells (ARPE-19) were treated with the following formulations: undiluted preserved TA (TA-BA), diluted preserved TA (D-TA-BA), preservative-free TA (TA-PF), and LB-VD. First, cell tolerability was evaluated with MTT, LDH, and ATPlite assays after 1, 5, and 30 minutes of exposure to each tested formulation. Then, cells were sequentially exposed to formulations and PFD. After 24 hours of exposure to PFD, cell tolerability was evaluated through MTT and ATPlite assays.

**Results:**

Among the formulations tested, LB-VD showed the highest levels of cell viability, cell metabolism, and cell proliferation and induced the lowest release of LDH, whereas the TA-based formulations demonstrated a cytotoxic effect on ARPE-19 cells in vitro. After subsequent 24-hour exposure to PFD, a greater reduction of cell viability was noted for all the formulations; however, this reduction was not significant only for the combination LB-VD-PFD, which was the best tolerated condition.

**Conclusions:**

LB-VD showed a better safety profile compared with all TA-based formulations, even when used in combination with PFD.

**Translational Relevance:**

In surgical practice, LB-VD may be preferred to TA-based formulations for vitreous staining in the light of its more favorable safety profile.

## Introduction

Vitreoretinal surgery is widely supported by the use of intraocular medical devices, such as intraocular tamponades and vital dyes.^1^^,^^2^ The safety of these compounds is a primary requirement for their use in surgical practice, and multiple regulations are aimed at this end.^2^ In particular, in vitro cytotoxicity tests are an essential part of the safety assessment process of these devices.^3^

Triamcinolone acetonide (TA) has been traditionally used off-label as an intraoperative intravitreal injection to stain the vitreous gel, improve its visualization, and thus facilitate surgical maneuvers such as the induction of posterior vitreous detachment, accurate removal of cortical vitreous remnants, and adequate trimming of the vitreous base.^4^ Indeed, TA is an aqueous dispersion of water-insoluble synthetic corticosteroid crystals that link the vitreous fibers, allowing their clear visualization.^5^ However, concerns have been raised regarding the safety of intraocular TA,^6^^,^^7^ and several experimental studies have reported the cytotoxic effect of TA on neuroretinal and retinal pigment epithelium (RPE) cells in vitro.^8^^–^^12^ The vehicle of preserved formulations and, in particular, the preservative benzyl alcohol (BA) have been investigated as the main causative factor of TA-associated cytotoxicity.^8^^,^^12^^–^^15^ Consequently, different strategies have been adopted to reduce the potential preservative-related toxicity, including the dilution of TA with balanced salt solution (1:3 or 1:4). Preservative-free TA formulations have been developed and registered as medical devices for intended intravitreal use; however, controversial results have been obtained with regard to their safety profiles.^9^^,^^15^^–^^17^

Recently, a lutein-based formulation designed for vitreous staining has been introduced as potentially safer alternative to TA-based formulations, offering some potential additional advantages related to the antioxidant, blue-light filtering, and retinal neuroprotective properties of this natural lipophilic molecule.^18^^,^^19^ Lutein-based blue dyes have shown good staining properties and favorable safety profile when used to stain internal limiting membrane (ILM) and/or epiretinal membrane (ERM);^20^^,^^21^ however, no studies have assessed the lutein-based vitreous dye (LB-VD) qualities.

Furthermore, it has recently been highlighted that the combined use of multiple intraocular medical devices during surgery could result in an additive and/or cumulative cytotoxic effect.^22^ Perfluorocarbon liquids (PFCLs), such as perfluorodecalin (PFD) and perfluoro-*n*-octane (PFO), are commonly used in the surgical management of complex vitreoretinal pathologies along with other intraocular medical devices, including vitreous dyes. Based on the above considerations, this study was designed to compare the safety profile of LB-VD and various TA formulations. Further, to mimic the intraoperative combined use of different medical devices, we evaluated cell tolerability following sequential exposure to these formulations and PFD.

## Methods

### Cell Culture and Experimental Design

Adult retinal pigment epithelial cells (ARPE-19; American Type Culture Collection, Manassas, VA) were cultured at 37°C (humidified atmosphere with 5% CO_2_) in Dulbecco's Modified Eagle Medium/Nutrient Mixture F-12 (DMEM:F12) medium (30-2006; American Type Culture Collection) and 100 U/mL penicillin, 100 μg/mL streptomycin, and 10% fetal bovine serum. The study was divided into two phases:

1. Phase I—Evaluation of the safety profiles of single-compound (LB-VD and TA) formulations on ARPE-19 cells with the following endpoints: cell morphology evaluation by 3-(4,5-dimethylthiazol-2-yl)-2,5-diphenyltetrazolium bromide (MTT) assay (Chemicon International, Temecula, CA), lactate dehydrogenase (LDH) release assay, and ATPlite (PerkinElmer, Waltham, MA) after 1, 5, and 30 minutes of treatment with formulations
2. Phase II—Evaluation of the safety profiles of the same formulations in combination with ultrapure PFD, with the following endpoints: MTT and ATPlite after 1 and 5 minutes of treatment with the formulations and 24 hours of treatment with PFD. We also attempted to evaluate the LDH release; however, we were not able to detect LDH, probably due to a strong interference with PFD in the medium.

### Compounds Tested

The compounds used for the test included the following:

• Undiluted preserved TA (TA-BA), A sterile aqueous suspension of TA at the concentration of 40 mg/mL (KENACORT-40; Bristol Myers Squibb, Lawrence Township, NJ). Each milliliter of the suspension contains 0.9% BA, 0.66% sodium chloride for isotonicity, 0.75% carboxymethylcellulose sodium, and 0.04% polysorbate 80.
• Diluted preserved (D-TA-BA), TA diluted with phosphate-buffered saline (PBS) at 1:3
• Preservative-free TA (TA-PF), Sterile injectable suspension for intraocular use containing 4% TA suspended in a saline solution at 7.2 pH with no preservative agents (Vitreal S; Fidia Farmaceutici S.P.A., Abano Terme, Italy)
• LB-VD, A 2% lutein suspension containing ultramicronized lutein crystals, povidone, and polyvinyl alcohol (VitreoLutein; Alfa Instruments, Arpino, Italy)
• Ultrapure PFD, density 1.93 g/cm^3^ (F-Decalin; Fluoron GmbH, Ulm, Germany)
• 1*H*-perfluorooctane (1H-PFO)—Molecular formula 1*H*,1*H*,2*H*,2*H*-Perfluoro-1-octanol (370533-5g; Sigma-Aldrich, St. Louis, MO)

### Cell Exposure

For the first set of experiments, after reaching confluence (∼80%), ARPE-19 cells were exposed for 1, 5, and 30 minutes to 50 µL of LB-VD, TA-PF, D-TA-BA, and TA-BA (the latter as positive control for BA^13^), without medium. In all experiments, only fresh medium was added in the control group (untreated cells, negative control). Potential cytotoxic effects were assessed with MTT, LDH release, and ATPlite assays. In the second phase, at cell confluence (∼80%), ARPE-19 cells were exposed for 1 and 5 minutes to 50 µL of LB-VD, TA-PF, D-TA-BA, and TA-BA, without medium. Then, the formulations were removed, fresh medium (300 µL) was added, and cells were exposed to 80 µL of PFD (83% of contact area), deposited directly on the cell layer. After a 24-hour incubation, the PFD bubble was removed, along with the medium. In this phase, 80 µL of 1H-PFO was used as positive control. Cell viability was assessed with MTT and ATPlite assays.

### Qualitative Cell Morphology Analysis

The cells were qualitatively evaluated using an inverted microscope (Leica DM IL LED; Leica, Wetzlar, Germany), and images were collected to assess cellular morphology after 1, 5, and 30 minutes of exposure to each tested compound. The morphological changes assessed included cell shape (regular/irregular, hexagonal, or smooth) and intercellular adhesion, related to the presence of the junction proteins.

### MTT Assay

The MTT assay was used to measure cellular metabolic activity as an indicator of cell viability, proliferation, and cytotoxicity. Optimal cell density was obtained by seeding 40,000 cells per well in 96-well plates (Costar; Corning, Inc., Corning, NY). Cells were exposed as previously described, and at the end of treatments, after washing with PBS 1×, ARPE-19 cells were incubated at 37°C with MTT (5 mg/mL) for 2 hours. Dimethyl sulfoxide was then added, and absorbance was measured at 570 nm in a microplate reader (Varioskan; Thermo Fisher Scientific, Waltham, MA). Results were reported as percentage of control. Graphs were built converting absorbance (abs) to viability (% of control) using the following equation: (abs*_x_* ÷ abs_ctrl−_)  × 100, where abs*_x_* is absorbance in the *x* well, and abs_ctrl−_ is the average absorbance of negative control cells (untreated cells).

### LDH Release Assay (Cell Permeability Index)

The LDH colorimetric assay is widely used for the evaluation of cellular cytotoxicity. The Invitrogen CyQUANT LDH Cytotoxicity Assay (Thermo Fisher Scientific) was used to measure the LDH cell release. ARPE-19 cells were seeded at 40,000 cells per well in Costar 96-well plates. After reaching confluence (∼80%), the cells were exposed as described above. After washing with PBS 1×, we added fresh medium. After 24 hours, 50 µL of medium was transferred into a new multiwell, and 50 µL of working solution was added. After 30 minutes at room temperature, 50 µL of stop solution was added. A Varioskan microplate reader was used to measure the absorbance values at 490 nm. LDH release was documented as LDH (% control) as follows: (abs*_x_* ÷ abs_ctrl+_)  ×  100, where abs*_x_* is the absorbance in the *x* well, and abs_ctrl+_ is the average absorbance of internal positive control cells (untreated lysed cells). Absorbance values were edited by removing blanks.

### ATPlite Assay

Cell viability was further assessed by measurement of adenosine triphosphate (ATP) production by means of the PerkinElmer ATPlite 1step Luminescence Assay System according to the manufacturer's protocol. After seeding and treatment as above, ARPE-19 cells were washed twice with PBS 1×, and 100 µL of buffer solution (ATPlite) was added to each well, according to the manufacturer's protocol. After 2 minutes of incubation at room temperature (shaker, 700 rpm), luminescence was assessed using the Varioskan microplate reader. Results were reported as percentage of control.

### Statistical Analysis

Statistical analysis was performed with Prism 7 (GraphPad, San Diego, CA). The data generated by all experiments were reported as mean ± SD (*n* = 6). One-way analysis of variance (ANOVA) was carried out, and Tukey's post hoc test was used for multiple comparisons. Differences between groups were considered statistically significant for *P* < 0.05.

## Results

### Phase I: Evaluation of Formulation In Vitro Cytotoxicity on ARPE-19 Cells

#### Cell Morphology Analysis

**Figure 1.**
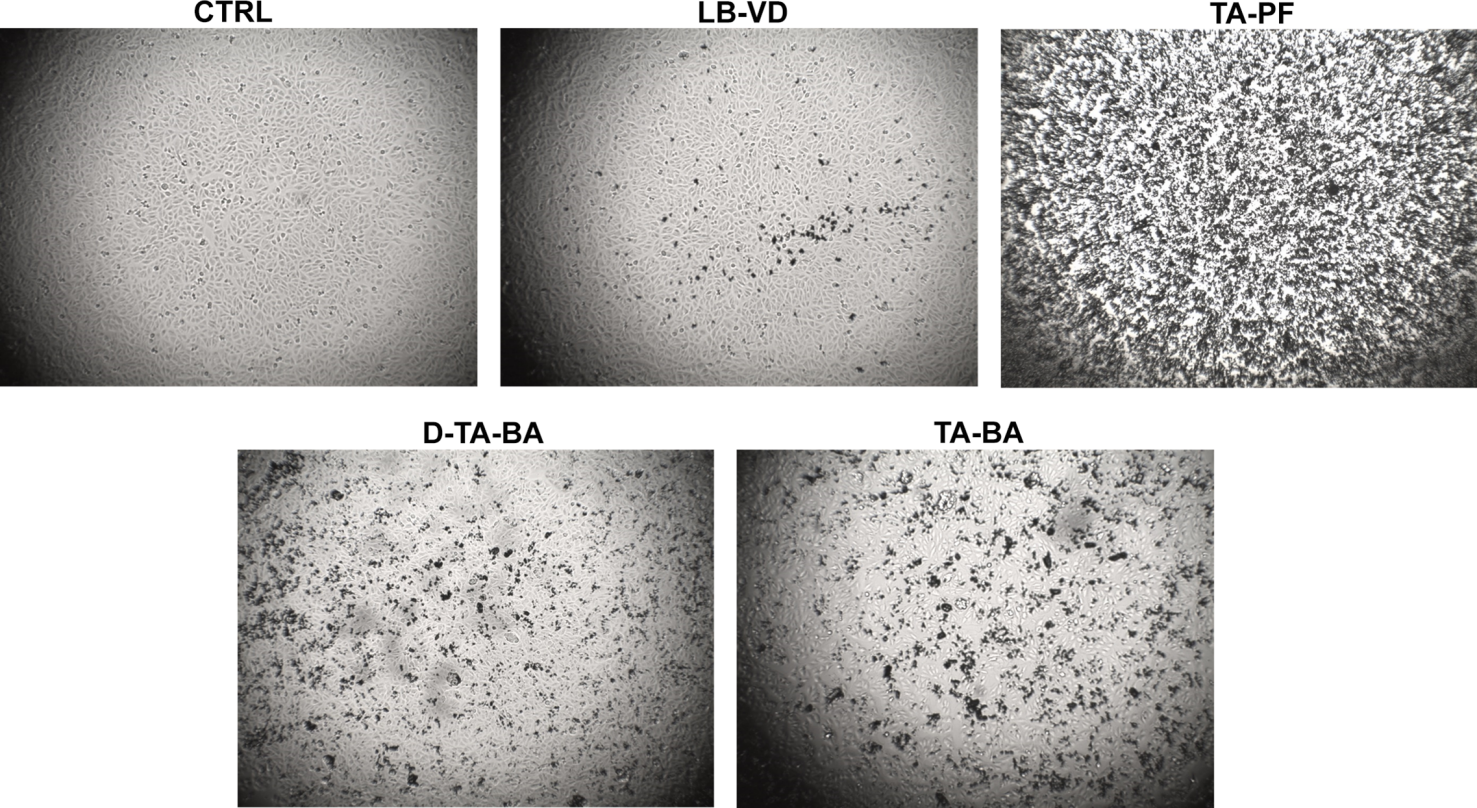
Representative images of ARPE-19 cells after 5 minutes of treatment with LB-VD (lutein-based formulation), TA-PF (preservative-free triamcinolone acetonide), D-TA-BA (diluted triamcinolone acetonide), and TA-BA (triamcinolone acetonide).

At all time points, no significant cellular damage was detected after treatment with LB-VD, and the epithelial layer was maintained. Exposure to D-TA-BA and TA-BA resulted in a significant decrease of cells number and adhesion (Fig. 1), with an evident cell morphology alteration (irregular shape). It was difficult to appreciate the ARPE-19 morphology after treatment with TA-PF, due to the TA aggregates deposited over the cell layer (Fig. 1). There were no significant differences in terms of morphological changes between the different time points.

#### MTT Assay

**Figure 2.**
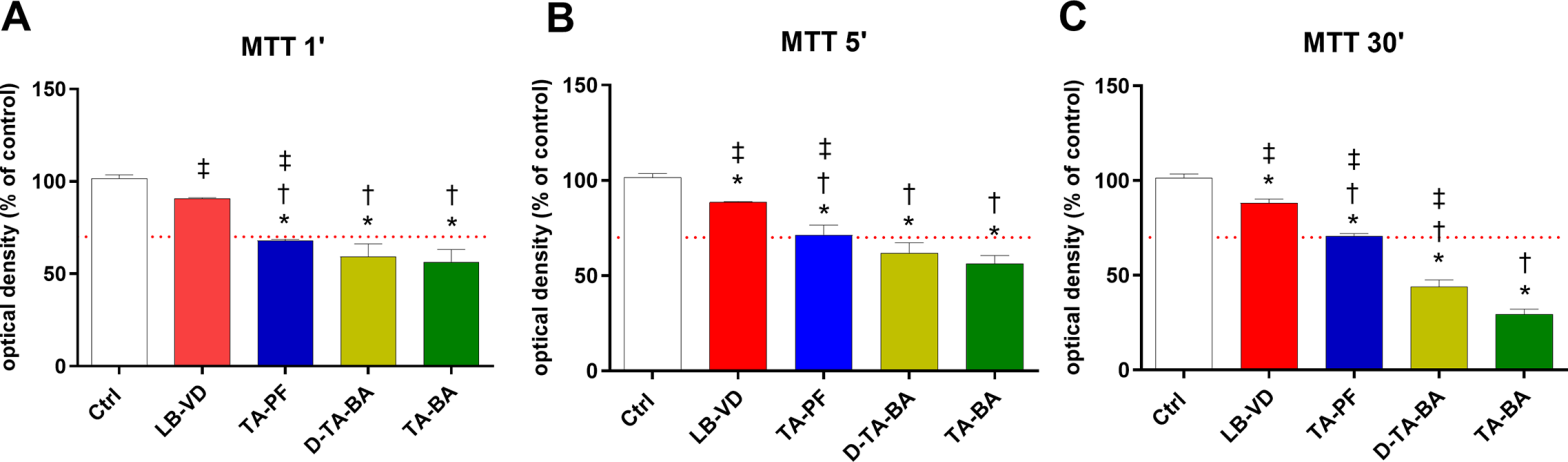
ARPE-19 cell viability after treatment with LB-VD, TA-PF, D-TA-BA, and TA-BA (ctrl+). *P < 0.05 versus control; †*P* < 0.05 versus LB-VD; ‡*P* < 0.05 versus TA-BA (ctrl+). Each bar represents the mean values ± SD (*n* = 6; each run in triplicate). Data were analyzed by one-way ANOVA, and Tukey’s post hoc test was used for multiple comparisons.

The results of the MTT assay are shown in the Table and Figure 2. LB-VD was the only compound that was not cytotoxic at all time points (Fig. 2), according to ISO 10993-5, 2009.^3^ The LB-VD induced a significantly lower cell mortality compared to all of the TA-based formulations (*P* < 0.05), regardless of the exposure time (Fig. 2). Preservative free-TA showed borderline cytotoxicity and had a significantly less impact on cellular viability than the preserved TA formulations (*P* < 0.05) (Fig. 2). Finally, both D-TA-BA and TA-BA were cytotoxic and induced a time-dependent reduction of cellular viability (Fig. 2).

**Table.** **Cell Viability as a** **Percentage** **of Control**^a^

	Mean ± SD								
	MTT			LDH			ATPlite		
Formulation Tested	1 Min	5 Min	30 Min	1 Min	5 Min	30 Min	1 Min	5 Min	30 Min
Medium (control)	102.1 ± 2.12	101.5 ± 2.12	100.1 ± 2.12	1.015 ± 0.02	1.012 ± 0.01	1.010 ± 0.01	101.0 ± 1.73	100.0 ± 1.23	101.5 ± 2.12
LB-VD	90.67 ± 0.57	88.59 ± 0.37	88.21 ± 2.04	1.025 ± 0.03	1.477 ± 0.02	1.348 ± 0.07	91.55 ± 1.22	88.10 ± 1.40	90.60 ± 1.12
TA-PF	67.88 ± 0.77	71.47 ± 5.13	70.68 ± 1.41	1.323 ± 0.04	2.024 ± 0.04	1.407 ± 0.03	83.00 ± 2.08	80.21 ± 1.47	83.80 ± 1.55
D-TA-BA	59.29 ± 6.93	61.96 ± 5.34	43.98 ± 3.53	1.359 ± 0.07	1.905 ± 0.11	1.684 ± 0.09	59.76 ± 1.32	54.61 ± 7.11	59.87 ± 2.82
TA-BA	56.41 ± 6.76	56.39 ± 4.19	29.38 ± 2.82	2.115 ± 0.02	2.935 ± 0.04	3.330 ± 0.21	38.17 ± 7.46	39.14 ± 5.87	42.25 ± 2.82

a The MTT, LDH, and ATPlite assays were used to measure cell metabolic activity and LDH and ATP levels in ARPE-19 cells.

#### LDH Release Assay

**Figure 3.**
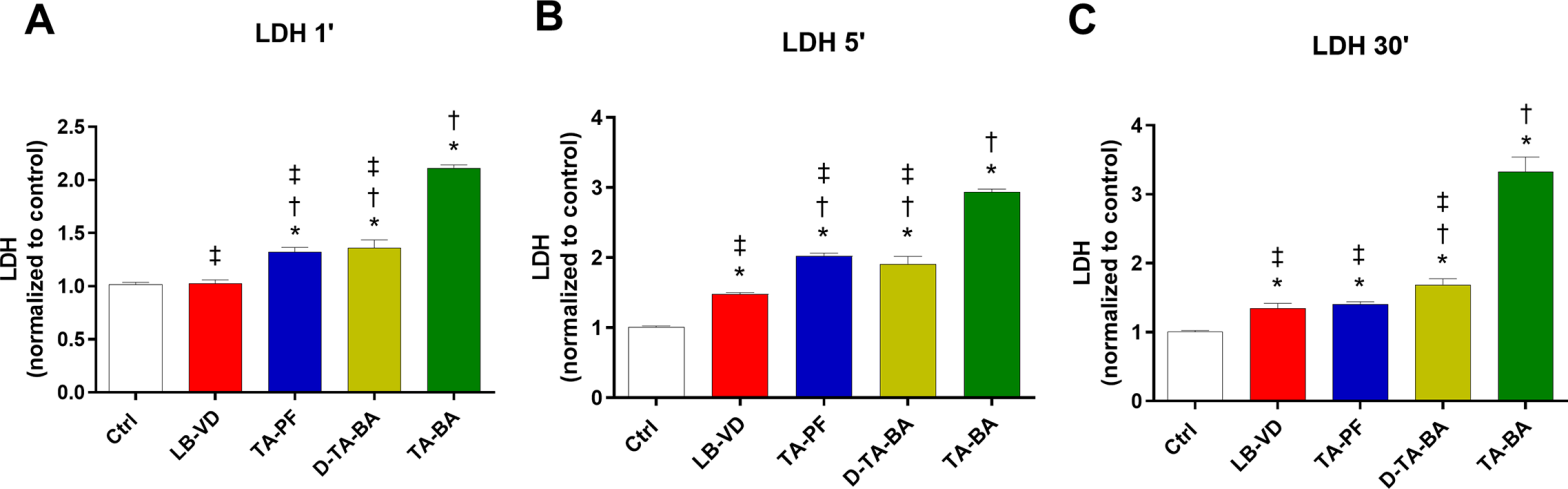
ARPE-19 LDH release after treatment with LB-VD, TA-PF, D-TA-BA, and TA-BA (ctrl+). *P < 0.05 versus control; †*P* < 0.05 versus LB-VD; ‡*P* < 0.05 versus TA-BA (ctrl+). Each bar represents the mean values ± SD (*n* = 6; each run in triplicate). Data were analyzed by one-way ANOVA, and Tukey’s post hoc test was used for multiple comparisons.

As shown in Figure 3 and the Table, treatment with LB-VD resulted in the lowest LDH release (*P* < 0.05), whereas TA-BA led to a significantly higher cell tolerance at all tested times when compared to the other compounds (*P* < 0.05).

#### ATPlite Assay

**Figure 4.**
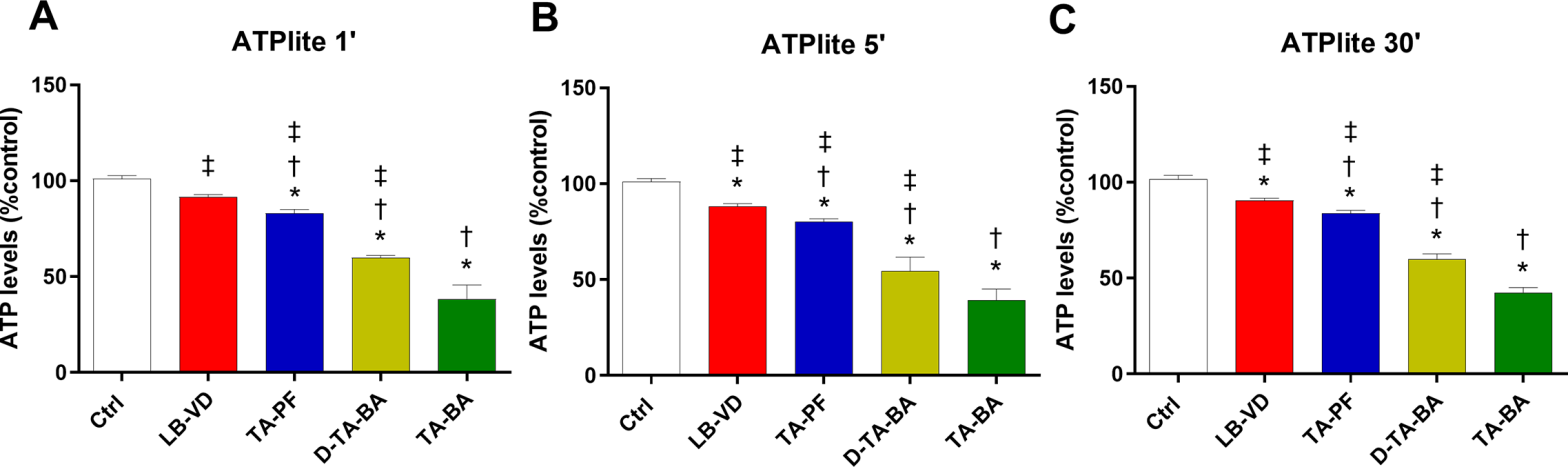
The ATPlite assay was carried out after treatment with LB-VD , TA-PF, D-TA-BA, and TA-BA (ctrl+). *P < 0.05 versus control; †*P* < 0.05 versus LB-VD; ‡*P* < 0.05 versus TA-BA (ctrl+). Each bar represents the mean values ± SD (*n* = 6; each run in triplicate). Data were analyzed by one-way ANOVA, and Tukey’s post hoc test was used for multiple comparisons.

In line with the MTT and LDH release assays, the ATPlite assay confirmed that LB-VD induced the lowest cell damage (higher ATP levels) when compared with TA-based formulations (*P* < 0.05). The decrease in ATP levels induced by TA-BA was significantly more pronounced in comparison to TA-PF and D-TA-BA (*P* < 0.05) (Table, Fig. 4).

### Phase II: Evaluation of In Vitro Cytotoxicity on ARPE-19 Cells After Sequential Exposure to Formulations and PFD

**Figure 5.**
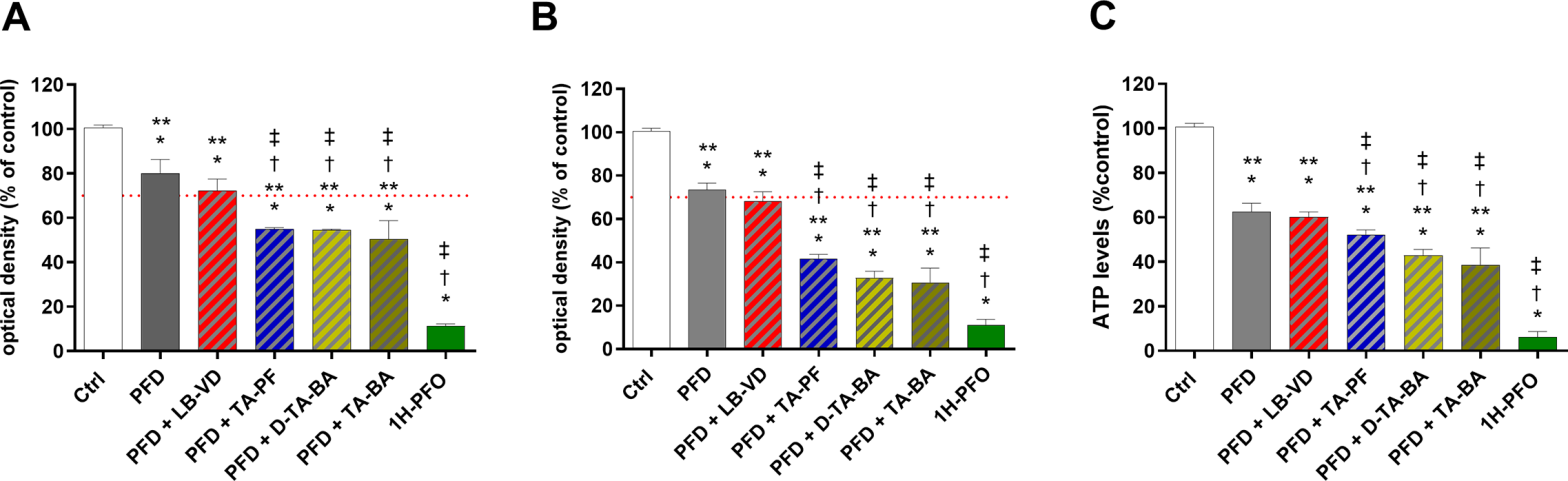
MTT and ATPlite assays after treatment with formulations and PFD. For all tests, ARPE-19 cells were first treated with the lutein-based formulations, TA-PF, D-TA-BA, or TA-BA. The formulations were then removed, and cells were treated with PFD and 1H-PFO for 24 hours. (A) The MTT assay was carried out after treatment for 1 minute with the formulations. (B) The MTT assay was carried out after treatment for 5 minutes with the formulations. (C) The ATPlite assay was carried out after treatment for 5 minutes with the formulations. *P < 0.05 versus control; **P < 0.05 versus 1H-PFO (ctrl+); †*P* < 0.05 versus PFD; ‡*P* < 0.05 versus PFD + lutein-based formulation. Each bar represents the mean values ± SD (*n* = 6; each run in triplicate). Data were analyzed by one-way ANOVA, and Tukey’s post hoc test was used for multiple comparisons.

**Figure 6.**
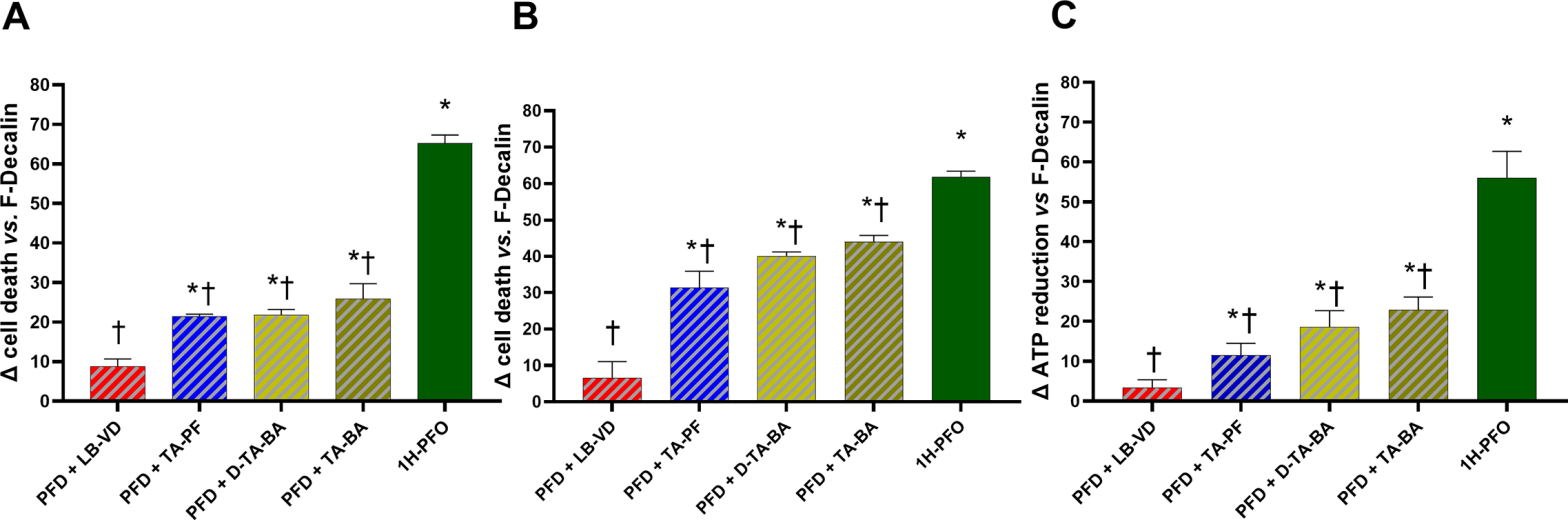
Delta values for cell mortality and ATP reduction after treatment with the formulations and PFD. For all tests, ARPE-19 cells were first treated with the lutein-based formulations, TA-PF, D-TA-BA, or TA-BA. The formulations were then removed, and cells were treated with PFD and 1H-PFO for 24 hours. (A) The MTT assay was carried out after treatment for 1 minute with the formulations. (B) The MTT assay was carried out after treatment for 5 minutes with the formulations. (C) The ATPlite assay was carried out after treatment for 5 minutes with the formulations. *P < 0.05 versus PFD + lutein-based formulation ; †*P* < 0.05 versus 1H-PFO. Each bar represents the mean values ± SD (*n* = 6; each run in triplicate). Data were analyzed by one-way ANOVA, and Tukey’s post hoc test was used for multiple comparisons.

Supplementary Table S1 summarizes the results of the experiments in this phase. Ultrapure PFD was not cytotoxic, despite exerting a negative effect on cell viability (Fig. 5). Following the sequential exposure to formulations and PFD, a greater reduction of cell viability was noted for all the formulations compared with the exposure to PFD alone. However, only in the case of LB-VD this reduction, was not significant (Fig. 5). Pretreatment with all TA-based formulations was associated with significantly greater cell damage when compared to PFD alone (*P* < 0.05) (Fig. 5). To better appreciate these differences, we calculated the delta values of cell mortality and ATP reduction, normalizing to only PFD-treated cells (Fig. 6). This confirmed that only LB-VD did not significantly impact cell viability and ATP production, in comparison to PFD-treated cells (Fig. 6).

## Discussion

Assessment of the safety profile of intraocular medical devices, such as vital dyes and ocular endotamponades, is a topic of growing interest in vitreoretinal surgery.^23^^–^^26^ In this regard, reliable methods such as in vitro cytotoxicity assays are useful for unveiling the potential toxic effects of these devices.^3^ In this study, we assessed the cell tolerability of a newly developed LB-VD in comparison to various TA formulations (TA-BA, D-TA-BA, and TA-PF) by means of direct-contact in vitro cytotoxicity tests on ARPE-19 cells, a validated testing method according to current regulations on intraocular medical devices, particularly ISO 10993-5, 2009.^3^ Furthermore, it has been previously highlighted that immortalized retinal cell lines, such as ARPE-19 cells, may be a more suitable and reliable alternative to primary retinal cells due to the high variability in the composition and quality of the latter based on the protocol and operator experience.^26^ Consistently, ARPE-19 cells have been largely used to study retinal cytotoxicity and the biocompatibility of vital dyes.^27^^–^^31^ Finally, the reliability of the results obtained using ARPE-19 cells has been supported by several previous studies evaluating the retinal cytotoxicity/biocompatibility of various intraocular medical devices. For example, comparable results have been documented when retinal ganglion cells and ARPE-19 cells have been used to assess cell viability after exposure to silicone oil (SO),^32^ as well as ARPE-19 cell and human retina ex vivo culture model to assess cell viability following exposure to non-cytotoxic PFCL samples.^23^ Based on this evidence, ARPE-19 cells may be considered appropriate to use to assess drug toxicity in retinal cells.

Over the last decades, various formulations of TA have been long used for the intraoperative staining of the vitreous gel and, less commonly, the ERM and ILM, during pars plana vitrectomy (PPV).^4^^,^^5^ However, concerns have been raised regarding the potential toxicity of TA. Several studies have demonstrated the in vitro cytotoxicity of TA, regardless of the presence or absence of preservatives in the formulation.^12^^–^^15^ LB-VD has been proposed as an alternative to TA due to its potential advantages in terms of safety profile. Lutein is classified as a natural dye by the U.S. Food and Drug Administration; in addition, the recognized antioxidant and blue-light filtering properties may help to prevent or mitigate potential phototoxic damage during PPV.^33^^,^^34^ Previous non-clinical and clinical studies reported promising results for lutein-based blue dyes in terms of safety and staining properties.^20^^,^^21^^,^^35^^–^^37^ Moreover, several in vitro studies have supported the protective effect of lutein on various types of retinal cells, particularly RPE cells, in response to different types of insult.^38^^–^^40^

However, to the best of our knowledge, no study has assessed the safety profile of LB-VD. We evaluated ARPE-19 cells treated with TA-BA, D-TA-BA, TA-PF, and LB-VD. The morphological analysis, regardless of exposure time, appeared to confirm the superiority of LB-VD in terms of cell tolerability, as it was the only compound not associated with significant morphological changes. Interestingly, TA-PF generated large aggregates that covered the ARPE-19 cells, thus hampering morphology assessment. This finding is consistent with a previous study that reported that a preservative-free TA injectable suspension (TAIS) was associated with larger aggregates compared with preserved TA.^41^ Further, Spitzer et al.^9^ showed that the size of TA aggregates correlated with cellular damage and death. Clinically, TA-PF may be more likely to create deposits in the most dependent part of the eye,^41^ thus resulting in a close and prolonged contact with the retinal tissue.

Regarding the quantitative examination, only LB-VD was not cytotoxic at all contact times, according to ISO 10993-5, 2009. Overall, LB-VD showed the maintenance of high levels of cell metabolism and proliferation, as demonstrated by the superimposable trends of the MTT, LDH release, and ATPlite assays. Conversely, TA-BA formulation showed cytotoxic effects, inducing a higher rate of cell mortality and damage. Moreover, we detected a time dependency in the cytotoxicity induced by preserved TA, both diluted and undiluted. This represents a crucial finding, as it has been demonstrated that preserved TA has a longer retention time in the vitreous cavity compared with different TAIS formulations.^42^ In surgical practice, the remarkable and time-dependent cytotoxicity of preserved TA can be relevant for multiple reasons. Indeed, TA can be detected up to 40 days after PPV.^43^^,^^44^ In addition, postoperative macular edema is a common complication after PPV,^45^ and the intravitreal injection of TA at the end of PPV for ERM has been proposed as an adjuvant for the prevention of macular edema, although no strong evidence supports this clinical use.^46^ In this regard, it has been demonstrated that TA may be more toxic when it is in direct contact with retinal tissue without the protective presence of vitreous (e.g., in the case of TA remnants, at the end of PPV).^9^

With regard to preservatives, the role of BA and vehicle in TA-induced toxicity is still controversial.^9^^,^^15^^–^^17^ It has been speculated that the vehicle induces retinal degeneration through both non-physiologic modification of vitreous pH (osmolarity or ionic composition)^13^^,^^14^^,^^47^ and exacerbation of TA cytotoxicity.^8^ Specifically, Chang et al.^14^ showed that BA induces RPE necrosis and triggers mitochondrial apoptosis. In our study, TA-PF had a better safety profile in comparison to TA-BA, but it induced cell mortality close to the cytotoxicity threshold of 30% (ISO 10993-5, 2009). On the one hand, the more favorable safety profile of TA-PF may support the role of BA and the vehicle as a contributing factor to the toxicity of preserved TA; on the other hand, the negative effect of TA-PF on cell viability and metabolism may reinforce the hypothesis of an intrinsic retinal toxicity of TA particles.

In surgical practice, multiple compounds can be used together or subsequently during the same procedure. For example, in the management of complex retinal detachment, PFCL and vitreal dyes can be used simultaneously to perform adequate vitreous base trimming, stabilizing the detached retina. It is known that different classes of medical devices can have physicochemical interactions. In particular, TA crystals in the presence of (SO) can form Pickering emulsions^48^^,^^49^; also, “sticky oil-like” solutions have been reported as the result of interactions between PFCL and heavy SO.^50^ Moreover, Gatto et al.^22^ speculated about a potential additive and/or cumulative cytotoxic effect due to interactions between different intraocular medical devices, as they showed that the simulated PPV in ex vivo porcine eyes resulted in a significant reduction of retinal viability when a visible amount of residues of TA, ERM/ILM blue dye, PFO, and SO were left intravitreally. For this reason, the use of selective dyes during ERM or ILM surgery can minimize the potential toxicity on retinal tissue.^51^ Based on the assumption that the interaction between different compounds may have to be considered to assess more reliably the potential harmful effect of the whole surgical procedure, we evaluated the in vitro cytotoxicity of sequential exposure to the formulations and PFD. Although in all of the experiments the combination of TA-based formulations with PFD reduced cell viability, sequential treatment with LB-VD and PFD was the best tolerated condition. Indeed, further studies are necessary to better investigate the effects and mechanisms of interactions between intraocular medical devices.

In conclusion, LB-VD exhibited the most favorable safety profile in comparison with various TA formulations. It follows that, clinically, despite their easy availability and good vitreous staining, TA formulations do not show a high safety profile. In addition, our results support the relevance of the interaction of different compounds in terms of potential impact on RPE cell viability. In this regard, LB-VD was found to be better tolerated in combination with PFD.

## Supplementary Material

Supplement 1
