# Supplementary material for: Safety Profile of Lutein- Versus Triamcinolone Acetonide–Based Vitreous Staining
Source: Transl Vis Sci Technol. 2023 Jan 4;12(1):5. doi: 10.1167/tvst.12.1.5 (PMC9832719; doi:10.1167/tvst.12.1.5)
Supplement: Supplement 1 [file tvst-12-1-5_s001.pdf]

Safety profile of lutein- *versus* triamcinolone acetonide-based vitreous staining

Francesca Lazzara[1], Federica Conti[1], Mariantonia Ferrara[2], Myrta Lippera[2,3], Michele Coppola[4], Settimio Rossi[5], Filippo Drago[1,6], Claudio Bucolo[1,6]*, and Mario R. Romano[3,7]

[1]Department of Biomedical and Biotechnological Sciences, School of Medicine, University of Catania, Catania, Italy; [2]Manchester Royal Eye Hospital, Manchester University Hospitals NHS Foundation Trust, Manchester, UK; [3]Department of Biomedical Sciences, Humanitas University, Pieve Emanuele, Italy; [4]Department of Ophthalmology, San Gerardo Hospital, Monza, Italy; [5]Multidisciplinary Department of Medical, Surgical and Dental Sciences, University of Campania "Luigi Vanvitelli", Naples, Italy; [6]Center for Research in Ocular Pharmacology–CERFO, University of Catania, Catania, Italy; [7]Eye Center, Humanitas Gavazzeni-Castelli, Bergamo, Italy

*Correspondence: Claudio Bucolo, University of Catania, Italy. E-mail: claudio.bucolo@unict.it

Table S1. The percentage values (% of control) of cell viability (optical density, MTT) and of ATP cell levels in ARPE-19 cells treated with formulations (1 and 5 minutes) and PFD for additional 24h, PFD only and 1H-PFO (positive control).

**Compound**	**MTT 1'**	**MTT 5'**	**ATPlite 5'**
**Ctrl**	100.7 ± 1.15	100.6 ± 1.05	100.8 ± 1.50
**PFD**	80.10 ± 6.30	73.40 ± 3.12	62.56 ± 3.76
**PFD + LB-VD**	72.36 ± 5.22	68.16 ± 9.73	60.14 ± 2.28
**PFD + TA-PF**	55.08 ± 0.55	41.52 ± 2.12	52.27 ± 2.06
**PFD + D-TA-BA**	54.69 ± 0.25	32.84 ± 3.07	42.85 ± 2.74
**PFD + TA-BA**	50.60 ± 8.19	30.61 ± 6.71	38.57 ± 7.66
**1H-PFO**	11.29 ± 0.93	11.10 ± 2.61	6.149 ± 2.48

LB-VD, lutein based vitreous dye; TA-PF, preservative-free triamcinolone acetonide; D-TA-BA, diluted preserved triamcinolone acetonide; TA-BA, undiluted preserved triamcinolone acetonide; PFD, ultrapure perfluorodecalin; 1H-PFO, 1-H perfluorooctane.
